# The schism in chiropractic through the eyes of a 1st year chiropractic student

**DOI:** 10.1186/s12998-017-0171-x

**Published:** 2018-01-16

**Authors:** Bob Strahinjevich, J. Keith Simpson

**Affiliations:** 1 Gosnells, WA Australia; 2 Discipline of Chiropractic, Murdoch University, Perth, WA Australia

**Keywords:** Chiropractic, Status of profession, Future trends, Vitalism, Biopsychosocial model, Organicism, Holism

## Abstract

Since its inception, the chiropractic profession has been divided along ideological fault lines. These divisions have led to a profession wide schism, which has limited mainstream acceptance, utilisation, social authority and integration.

The authors explore the historical origins of this schism, taking time to consider historical context, religiosity, perpetuating factors, logical fallacies and siege mentality.

Evidence is then provided for a way forward, based on the positioning of chiropractors as mainstream partners in health care.

## Background

Asking a deceptively simple question such as what is chiropractic inevitably results in a complex answer. Perhaps at the very core of this complexity is the fact that chiropractic is a divided profession, plagued by internal and external conflicts.

Internally, the divergences have manifested themselves as an identity struggle, with many chiropractors seeking a moderate, evidence based position while others strive to retain vitalistic ideas [1, 2]. The disparity between these groups has divided the profession and invited ridicule from the both the scientific community [3] and the public at large [4]. Additionally, disagreements around scope of practice [5], vocabulary [6] and ethics [7] have negatively affected public opinion [8], cultural authority [6, 9] and inter-professional relations [3]. Externally chiropractic has been embroiled in conflict with political medicine practically since chiropractic was ‘discovered’ in 1895 [6].

Over several decades, researchers have sought to determine the origins of these conflicts [10–13] and offered solutions [6, 14–17]. Condensed to simplest principles, the division reflects a deep ideological gulf which has historically been described as the schism between ‘mixers and straights’ with acceptance or rejection of treatment modalities other than ‘the adjustment’ as the dividing point [1, 10, 11, 18]. This however is an overly simplistic and patently misleading understanding.

Phillips framed the schism more accurately and succinctly around “believers and questioners”: those who believe the foundational vitalistic premises of Innate Intelligence (II) and Universal Intelligence (UI) should act as the guiding light of chiropractic versus those who question the relevance of basing patient care on unverifiable, a-priori assumptions and importantly, the role that science plays in both factions. For believers science is explanatory whereby science will prove what believers know. That is, “beliefs are based on evidence derived from observations that support the universal, the Major Premise”. This is in contrast to questioners for whom science is investigatory – “a search for understanding and clarification of what it is that chiropractors do, and determine if it is effective”. [19] p4.

Unfortunately, entrenched ideologies, based on a misunderstanding of science and marinated in the fear of losing a ‘separate and distinct’ (from all things medical) identity [20] have prevented chiropractic from fully unifying and moving forward. Ultimately, progress can only come about from a shared, scientifically sound vision.

This paper had its origin as an essay assignment by a first year chiropractic student (BS). This expanded version of the essay will review the historical origins of the schism in chiropractic (schism) and examine the influence the schism has had on the profession. It will consider the many reasons that the schism has persisted for over a century and discuss a possible strategy whereby the schism could be healed, and the chiropractic profession take its justified place within the twenty-first century health care system.

## Chiropractic philosophy versus philosophy of chiropractic: ideology, religion and history

In simplest terms, the schism came about as result of a mismatch between dogma and scientific progress [21] or as Donahue correctly asserts: the difference between chiropractic philosophy which stresses philosophy as a doctrine and philosophy of chiropractic which recognises philosophy as an activity [22].

Martin [11] reminds us that chiropractic arose in the United States of America (USA), at a time of both rapid industrial change and a time when there was no conflict between science and religion. Martin also points out that the prevailing worldview in the late 1800s was one that linked health care to a broad philosophical base in which a benevolent God ruled the universe through natural laws. Chiropractic emerged into this milieu as an amalgamation of vitalistic and harmonial religious philosophies which were envisioned by their adherents, of which DD Palmer, the ‘Discoverer of Chiropractic’ was one, as alternatives to Christianity [23]. Ahlstrom [24], in his definitive analysis of religions in the USA, categorized harmonial religions as those forms of religion in which spiritual calmness, physical health, and even economic well-being are understood to flow from a person’s oneness with the universe. Importantly for this discussion harmonial religions were challenging Judaism and Christianity. Harmonial religions may be more readily recognized by their somewhat pejorative labels including: healthy-minded religions, women’s religions, metaphysical religions, mind-cure religions, and positive thinking religions [25].

There is no doubt that DD Palmer’s early chiropractic had a distinct religiosity to it and this was not diminished when BJ Palmer, the ‘Developer of Chiropractic’ took over the reins from his father, DD Palmer. Most certainly the separation between Christianity and Chiropractic grew under BJ’s directorship, however the religiosity of chiropractic did not. [26] Further, chiropractic, being based in part on harmonial religious and vitalistic ideas [27] initially rejected advancing medical science in favour of the doctrine of the ‘healing power of nature’ (vis medicatrix naturae). The intuitive simplicity of vitalism no doubt appealed to the laity in the American heartland, who empirically understood the self-repairing nature of the body, tended to be deeply religious and viewed advancing medical technologies with skepticism [11].

As such, espousing vitalism would have benefited early chiropractors, both in terms of commerce and professional unity. However, elements within the theory would soon cause a profession wide rift [14].

## The problems with UI, II

After ‘receiving’ the principles of chiropractic from the spirit of Dr. Jim Atkinson [28], DD Palmer developed his theory to include the healing power of nature (in the form of II), a key principle in Palmer’s formulation, which embodied “the religious plank of the foundation of Chiropractic” [28] p. 642. Palmer the elder is on record as stating that 95% of all diseases could be attributed to misaligned vertebrae, which impaired the flow of II within the body while Palmer the younger (BJ Palmer, DD’s son) was of the opinion that 100% of all disease was caused by vertebral misalignment, later known as subluxation [29]. Importantly for the chiropractic cause, chiropractic subluxations, being different from medical subluxations, could only be identified and repositioned by the skilled hands of a chiropractor [30].

UI and II, couched in supernatural terms, were viewed as manifestations of God’s natural laws acting upon the body [11]. DD was clear in his writings:

Innate is part of the all wise. Innate is a part of the Creator. Innate spirit is a part of Universal Intelligence, individualized and personified. [28] p. 691

God -the Universal Intelligence- the Life- Force of Creation. [28] p. 446

The Palmers (DD and later BJ) saw UI and II as inviolate articles of faith, so much so that DD recommended “hoisting a religious flag” and seeking legislation for the right to practise the religion of chiropractic with he, DD Palmer, as the religious head similar to “Christ, Mohamed, Jo. Smith, Mrs. Eddy, Martin Luther and other [sic] who have founded religions” [31]. Religiosity within early chiropractic leaders is abundantly evident:

- DD Palmer wrote: ‘I believe, in fact know, that the universe consists of Intelligence and Matter. This intelligence is known to the Christian world as God’ … a correct understanding of these principles and the practice of them constitute the religion of chiropractic [28]
- BJ Palmer compared himself to Jesus Christ and being crucified by medical opposition [32]
- Chiropractors who were jailed for practising medicine without a license were referred to as martyrs [11]
- BJ Palmer referred to Nugent, an advocate for improved academic standards within chiropractic, as the anti-Christ of Chiropractic. [32]

**Fig. 1:**
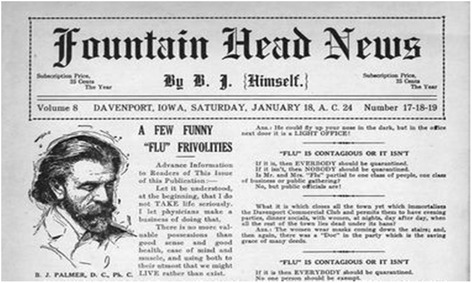
Fountain Head News January 12, A.C. 24

Further indications of the religiosity of BJ Palmer are apparent when, in 1916 he brazenly revised the Gregorian calendar to denote 1895, the year chiropractic was discovered, as year zero. Thus 1916 became AC 21 for After Chiropractic 21. BJ used this notation on the masthead of every issue of the Fountain Head News (FNH) in an effort to remind readers that chiropractic was as significant as religion and that its discovery date paralleled in importance the birthdate of Jesus Christ [20]. (See Fig. 1) BJ continued this practice until his death in 1961.

This faith-based position led to pseudoscientific and anti-scientific tendencies [33] that still permeate the profession.

For Palmer with his belief in vitalism and spiritualism, the only explanation for observations of the healing capacity of the body was a supernatural one, thus UI and II came into being. Unfortunately, DD Palmer’s conclusions about Innate Intelligence greatly overreached the scientific knowledge of the era [27]. To put this into perspective, Bernard’s concept of internal stabilisation of the body arose from the mid-1800s with the word homeostasis not arriving into the physiology lexicon until Cannon coined it in 1926 after which Cannon’s publication of The Wisdom of the Body in 1932 made ‘homeostasis’ a household word [34].

For his part, BJ seized upon UI and II with evangelical zeal proclaiming “Get the Big Idea [The idea that knows the cause, that can correct the cause of dis-ease, is one of the biggest ideas known] and all else follows” [20] p177.

As if to compound and reinforce pseudoscientific matters, Ralph Stephenson, a 1921 PSC graduate and member of the Palmer ‘philosophy’ faculty, wrote The Chiropractic Textbook. The book goes into great detail on chiropractic and consolidates the ideas described throughout what Senzon labelled as the Collaborative Phase of philosophy (1916–1926) [35]. It was the first publication of The 33 Principles of Chiropractic.

Although they are still debated and cast in metaphysical terms, The 33 Principles are nevertheless used today by many chiropractors as a source of philosophical inspiration and professional identity [36, 37].

Stephenson’s text was endorsed by BJ and used as the primary Chiropractic Philosophy text at PSC and all other ‘believer’ schools [20].

Aside from a misuse of the word philosophy [38] there are clear indications that Stephenson did not appreciate basic science principles. This is exemplified by his declaration:

Deductive reasoning is exactly suited to Chiropractic. **By assuming a major premise**, that there is a Universal Intelligence which governs all matter, every inference drawn from that major premise and subjected to specific scrutiny, stands the test. [Emphasis added]. [39] p. xx

A further indication that Stephenson’s understanding of the role of science was wanting is embodied in his statement:

Chiropractic reasons deductively instead of inductively, accepting scientific findings with every finding being more proof of its Major Premise. [39]. p. xx

In other words, science will prove what believers already know: ‘Chiropractic Works!’ [40].

Further, Stephenson renounced inductive reasoning as reductionist and associated with medicine [19, 39], with whom chiropractors shared an adversarial history.

One early prospectus for Palmer School of Chiropractic (PSC) sums up the position nicely –

We do not waste valuable time in observing healthy and morbid tissue under the microscope… students save time and money by omitting these useless studies. [41]

Concurrently, medicine made great strides to standardize its education and began to adopt biologically plausible explanations for disease and methods of treatment [42], thereby gaining cultural authority. This placed chiropractic and medicine at loggerheads, leading to chiropractic being labelled quackery and calls for the imprisonment of chiropractors on the grounds of practising medicine without training or licence [43, 44].

In essence, the very principles (UI and II) that served to cement chiropractic in the popular consciousness would later cause a rift between it and organised medicine. Furthermore they would drive a wedge between progressive, scientifically oriented chiropractors and fundamentalists who sought to maintain the status quo. Ultimately, UI and II would prove to be a mixed blessing.

## A mixed blessing

As chiropractic grew, the Palmerian anti-scientific dogma began to be regarded by many early ‘disciples’ as religious baggage, which began to chafe questioners within the profession to the point of taking decisive action. This was fully evident by 1906 just a few short years after DD opened the Palmer School of Chiropractic in Davenport and started teaching his techniques in 1897. Some of the key departures from the Palmer path are touched on here.

Opening schools to teach chiropractic was not unusual for Palmer graduates. Indeed, this was in keeping with the instruction to “teach and practice” chiropractic as noted on Palmer’s certificate of graduation [45]. In 1901, the same year that he graduated from Palmer’s school, Solon Langworthy, one of DD Palmer’s first 15 disciples, established a more contemporary school: Langworthy’s Cedar Rapids [Iowa] Chiropractic School and Cure. Langworthy integrated other treatment methods in his approach, including osteopathy, naturopathy, medical orthopaedics, as well as the use of mechanical traction and stimulation devices [46].

Later, Oakley Smith and Minora Paxon, both Palmer graduates and faculty members of DD’s Santa Barbara California school, joined Langworthy’s faculty and in 1903 his school was renamed the American School of Chiropractic & Nature Cure (ASC).

At this point Langworthy proposed a partnership with the Palmers that would see the ASC combine with the Palmer schools and others opened. While BJ was open to the idea, DD made clear in his rejection in his letter to Langworthy.

Chiro. [sic] is not benefited by mixing it with any other method [46] p. 5

Those who desire to practice it with other methods have a right to do so, but if they call the mixture chiropractic we will call them down. [46] p. 6

DD’s resounding rejection of the prospect of combining chiropractic with various other healing procedures was the beginning of what is under scrutiny here: the straight [pure]/mixer schism with Langworthy arguing that Palmer’s concept of adjustments by hand was not sufficiently scientific and accurate and DD staunchly arguing that chiropractic was done by hand only [20].

Langworthy’s influence on the fledgling profession is noteworthy for the number of firsts he achieved. The ASC was of particular importance because it was the first chiropractic school to establish a systematized curriculum of lectures and clinical work. Langworthy published the first regular scholarly chiropractic journal – The Backbone – and, with co-authors Smith and Paxon, published the first chiropractic textbook – Modernized Chiropractic. Also, Langworthy established the first organized chiropractic society, the American Chiropractic Association. [45] Oakley Smith, Langworthy’s associate and co-author introduced the word ‘subluxation’ into the chiropractic lexicon as well as the concept of the intervertebral foramina being involved with interference with nerve transmission [45] and importantly to the believer/questioner divide, Langworthy argued that the brain was the source of the “Unseen power” that gave force to the nerves, not Universal Intelligence as espoused by DD. [47]

In addition Langworthy was instrumental in securing the passage of the first chiropractic legislation to regulate the practice of chiropractic in the USA. The 1905 Act stipulated required examinations in courses similar to those taught at the American School and specified that applicants required 2 years of training in an approved school of chiropractic in order to be licensed. Not only did this mean that the standard of chiropractic education was mandated and uniform, it meant that he and his graduates were able to practise chiropractic with impunity from prosecution. While this might have been regarded as a significant achievement for many in chiropractic, it enraged DD who lobbied the Governor and the bill was vetoed but the cracks within chiropractic remained [20] pp37–8.

Willard Carver, a lawyer and chiropractor was a staunch supporter of DD. Indeed, he had pleaded DD’s case for a pardon with the state Governor following DD’s 1906 conviction for practising medicine without a licence. By this time however, Carver was dissatisfied with BJ’s leadership and no longer content to continue to do business with DD because of “his particular impulsiveness and his strange, not to say erratic way of doing things” [48] p.19 and in the same year formed the break-away Carver College of Chiropractic in Oklahoma City [14] naming it as the “science-head of chiropractic” to distinguish it from Palmer’s “Fountain Head”. Carver continued his foray into chiropractic education by opening schools in New York City (1919), Washington DC, (1922), and Denver Colorado (1923). Carver’s curriculum rejected DD’s concept of Innate Intelligence healing the body, favouring a naturalistic view in which physiologic processes reconstruct the body [47].

By 1906 John Fitz Alan Howard – a one time faculty member at PSC – was well aware of the rampant dissent at Palmer School of Chiropractic. That same year Howard became so disturbed by the low level of scientific literacy among PSC graduates that he moved, with DD’s approval, to form a new, scientifically rigorous school in the same building where DD had begun his School and Cure in Davenport Iowa. [29] In 1908 Howard’s National School of Chiropractic was moved to Chicago where it employed medical doctors to teach anatomy, chemistry and diagnosis, thereby setting Howard at odds with PSC [10]. Further, Howard’s warning to students not to “dwindle or dwarf chiropractic by making a religion out of a technic” [29] p.17 did little to ingratiate him with the Palmers.

These are but a smattering of the examples of the ideological wars that were raging within the new chiropractic profession. While the initial adherence to vitalistic ideas solidified chiropractic, the Palmers’ leadership – with their adherence to vitalism and marginalization of science – stymied progressive thinkers and derailed mainstream integration. This led to an endless feedback loop between questioners and believers, each entrenching the other’s position in the phenomenon identified by Tourangeau and Rasinski as the ‘backfire effect’ [49].

To date, neither side has been able to find true common ground [1, 6], often using arguments and tactics that have increased division, further split the profession and negatively affected social standing [50].

## The hundred years’ war: what perpetuates this ideological dispute?

While not as interesting as the conflict between the Houses of Plantagenet and Valois, the question of why the schism has persisted for a century is, from an insider’s perspective, perplexing and from any observer external to the profession, fascinating. At the risk of offending, we suggest that the answer lay in a blend of facts, alternative facts, and logical fallacies, each of which will be explored below.

The facts regarding the schism are fairly straightforward and have, to a large extent been examined in the preceding sections.

Whether the commonly portrayed events surrounding the ‘discovery of chiropractic’ in 1895 are facts or alternative facts is not so clear. According to DD Palmer, a well-read, self-educated magnetic healer, chiropractic was ‘discovered’ on September 18th 1895 during the pre-scientific era of health care. [51] Palmer claimed to have restored the hearing of Harvey Lillard, the deaf janitor in the building where Palmer had his magnetic healing clinic. According to Palmer his first adjustment was not a chance affair. Rather, it was a calculated intervention and the results were not unexpected. [51, 52]

There is however, credible evidence that the famous adjustment did occur not in the manner described by Palmer [52] on the claimed date of 18 September 1895. Indeed, the best guess is that chiropractic was ‘discovered’ somewhere between September 1895 and January 1896 [53]. There is even disagreement on what section of Lillard’s spine was adjusted [20]. Giving Palmer the benefit of the doubt, it is perhaps safer to refer to his account of the Lillard incident as what Donald Trump describes as ‘truthful hyperbole - an innocent form of exaggeration — and a very effective form of promotion’ [54]. p 58.

Whatever its origins, the facts are that chiropractic has gone on to be widely recognised as the third largest primary contact health care profession in the Western world with millions of ‘satisfied’ clients attending for care every year [55] – proof positive that ‘chiropractic works’. This is but one of chiropractic’s fallacies^1^: argumentum ad populum. It is fallacious to argue a premise [chiropractic works] on the basis that it must be true because so many people believe it or use it.

Chiropractic emerged in the late 1800s towards the end of the Era of Heroic Medicine. During most of the 1800s three groups of healers, few of whom had much in the way of formal training, dominated the health care system: the eclectics, homeopaths and regular physicians [56]. Many health care providers, including DD Palmer, were searching for what might be considered the magic bullet for mankind – the cure for all of mankind’s ills.

While DD Palmer had no formal training, he was an avid reader of medical journals, which provided him with an in-depth understanding of anatomy and physiology, as it was known in the late 1800s. Analysis of his writings suggests that his grasp of these sciences may well have been more extensive than that of his medical contemporaries [22, 57, 58] and Palmer’s explanations for chiropractic were derived from the concepts of the neurology current in the late 1800s [59].

DD Palmer believed he had discovered, not just the magic bullet but the answer to the question: what is life? DD’s ‘discovery’ may well have been the first of chiropractic’s three initial fallacies. The discovery was The Fallacy of the Crucial Experiment: claiming that some idea [chiropractic subluxation theory] has been proved by a pivotal discovery [Lillard’s restored hearing]. NB: this is disregarding the biological implausibility of the claim. The second was The Golden Hammer Fallacy: Proposing the same type of solution [adjustment] to different types of problems [all of mankind’s ills]. And the third was The Argument to the Future: Once the answer was known – chiropractic – it was only a matter of time before proof would emerge confirming what the enlightened already knew.

When BJ Palmer took over the reins of the Palmer School of Chiropractic in 1903 he ruled with an iron fist [20]. Much of what occurred during BJ’s era at the helm of The Fountain Head was driven by BJ’s adherence to Blind Authority Fallacy: Asserting that a proposition [The Big Idea] is true solely on the authority making the claim while also ignoring any counter evidence no matter how strong and Proof by Intimidation: employing intimidation to prevent questioning the authority or a priori assumptions of the one making the argument.

While these approaches may be compelling, they did not encourage enlightenment for the developing profession. Rather, we have argued that this was the cause of the 100-year schism or, as Keating phrased it, perpetuating a state of static rationalism versus dynamic science [60] p.81. DD himself would likely have found this state of affairs perplexing as indicated by his statement on “classes of chiropractors”.

There are two classes of Chiropractors, those who desire to know all they can of physiology, pathology, neurology and anatomy, and those who have an aversion for intelligence, do not want to take effect into consideration, depending only upon an examination of the spinous process. (Palmer 1910) p. 335

Prior to 1910 there was little drive for health care reform. The impetus arrived in the form of Abraham Flexner’s 1910 report [61] which was heavily promoted by an organized American Medical Association [62].

While King argues that Flexner’s report was “probably the most grossly overrated document in American medical history” [63] p.1079, others hold quite a different position and there is no doubt that the Flexner Report heralded the Golden Age of Doctoring and its associated medical dominance of the health care system [64]; a dominance which would continue for decades [65]. The Golden Age represented a move to scientific medicine and concurrently a rejection of the sectarian health care system that the Golden Age of Medicine replaced. Furthermore, there was a move to professionalism within medicine that brought with it an eschewing of advertising [64].

The Flexner Report also facilitated the demise of virtually all other healing sects [66], with the obvious exception of chiropractic. In a damning statement, Flexner made his considered position on chiropractic abundantly clear. Chiropractic did not deserve mention in any educational discussion. Rather,

The chiropractics [sic]. the mechano-therapists, and several others are medical sectarians, though exceedingly desirous of masquerading as such; they are unconscionable quacks, whose printed advertisements are tissues of exaggeration, pretense and misrepresentation of the most unqualifiedly mercenary character. The public prosecutor and the grand jury are the proper agencies for dealing with them. (Flexner 1910) p.158

The court system did its very best to stem the growth of chiropractic. Within the first 30 years of the chiropractic profession’s existence there were more than 15,000 prosecutions for practising medicine without a licence, about 20% of which resulted in incarceration [67]. However, it was to no avail. As stated above, the fledgling profession grew into the third largest primary contact profession in the Western world albeit an ideologically divided one. What did emerge from the thousands of prosecutions was a mantra, one that played an effective role in the thousands of courtroom proceedings:

- Chiropractic is a separate and distinct ‘philosophy’.
- Chiropractic is the antithesis to all things medical.
- Chiropractors do not diagnose; they analyze the spine for subluxations.
- Chiropractors do not treat any disease: they remove subluxations and thereby remove the interference caused by subluxations.
- Only a body free of subluxations can reach its full potential. [20]

This mantra, believed by many and accepted by most, became a part of the chiropractic psyche and irrevocably part of chiropractic culture [40].

A separate and distinct chiropractic professionalism also emerged, one that differed substantially from traditional professionalism. While it is widely accepted that altruism is central to traditional professionalism [68], chiropractic professionalism favoured entrepreneurialism and recognised financial success as a strong indicator for accomplishment [11].

In stark contrast to the medical profession’s ethical ban on advertising, chiropractic embraced marketing. Even chiropractic students were encouraged to advertise heavily [11, 69].

**Fig. 2:**
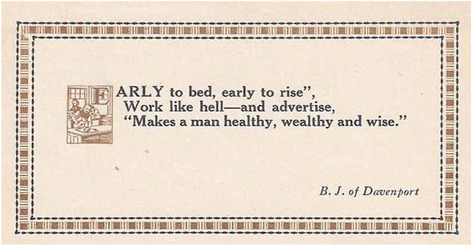
BJ Palmer’s Advertising Epigram

This was epitomized by BJ’s advertising epigram^2^ (See Fig. 2):

This chiropractic professionalism did nothing to endear it to other more powerful players in the health care system. Further, the apparent rejection of traditional professional attributes in favour of free-market principles was, and continues to be a source of internal friction within the profession. [70–72].

In conjunction with the years of prosecutions through the legal system, the fledgling chiropractic profession endured decades of persecution with the implementation of the American Medical Association’s Iowa Plan which sought to ‘contain and eliminate chiropractic as a health hazard in the United States’ [and abroad] [73].

These external influences were enough to unite the profession in a common defence, a necessary contributor to its survival. The downside of the common defence was the resultant development of a siege mentality: a belief held by group members that those outside the group have negative behavioural intentions towards them (Bar-Tal and Antebi 1992). A siege mentality is strengthened by what Bar-Tal describes as delegitimization whereby a group is categorized into extremely negative social classifications and thereby excluding them from acceptability (Bar-Tal 1990).

Given the prolonged history of chiropractic persecution and prosecution and the Iowa Plan’s labelling of chiropractic as a hazard to rational health care and chiropractors as rabid dogs and killers [73] it would be difficult to argue that chiropractors were incorrect in holding a belief that others had negative behavioural intentions toward them. I.e. a siege mentality developed as a part of the culture of chiropractic.

Bar-Tal identified several characteristics of a culture with a siege mentality (SM) and importantly for this discussion, Bar-Tal highlights 4 consequences of a siege mentality [74]. These are:

1. The SM group develops extremely negative attitudes towards other groups.
2. The SM group develops extreme sensitivity to any actions by other groups that may be perceived as negative toward the SM group. There is distrust and suspicion on the part of the SM group toward any ‘opposing’ group.
3. The SM group develops internal mechanisms to ensure conformity and obedience to its ideology.
4. The SM group may take actions that are out of step with internationally accepted behavioural codes.

It is beyond the scope of this paper to perform a comprehensive analysis of chiropractic with regard to Bar-Tal’s siege mentality consequences however there is sufficient evidence already published to argue in favour of their acceptance [40, 75–78].

Arguably political medicine’s campaign against chiropractic was impetus for a siege mentality within chiropractic [79]. When the anti-chiropractic campaign effectively ended with the Getzendanner decision [80] in the USA and the decision of the Australian Consumer and Competition Commission in Australia [81, 82] an era of interprofessional cooperation the likes of which the chiropractic profession had never experienced began [83] and continues today.

With the majority of the schism’s perpetuators removed or resolved, one might expect the schism to disappear. Sadly this is not the case. The schism continues to exist perhaps because of “fear of extinction from the onslaught of modernity” [78] p. 9 and all that entails and furthermore, the schism continues to have a negative impact on the profession. This raises the ‘so what’ question: should chiropractic become mainstream? It has survived largely on its own for this long; perhaps remaining separate and distinct is a good thing?

## Should chiropractic become mainstream? What are the benefits?

Even though chiropractic has earned many of the features of a mainstream healthcare profession [18], vitalistic groups within chiropractic have long sought to maintain a ‘separate and distinct’ [from all things medical] footing [84]. By doing so, they have stymied progress towards integration [21, 85].

As discussed in previous sections, this position – which appears to have arisen due to stubborn adherence to vitalistic ideology – has limited acceptance, hindered inter-professional cooperation within health care and brought ridicule from both the academic and scientific communities [86]. Perhaps worst of all, it has perpetuated a subculture of unethical practice, negatively impacting cultural authority [7, 72] and limiting utilisation. [3, 87]

Given this state of affairs, are there benefits in transforming and integrating chiropractic within mainstream health care? We suggest the answer is a resounding yes.

Firstly, closer collaboration within mainstream health care represents a benefit to society. By dint of training, chiropractors are uniquely placed to offer conservative, non-surgical management of spinal conditions. This places chiropractors at the forefront of addressing musculoskeletal disorders in general and spinal pain specifically.

Given that chronic low back pain represents the second leading cause of disability world-wide [88] – and that chiropractic appears to be a safe, effective and cost effective intervention [89] - positioning chiropractors as a mainstream partner addresses a shortfall within the health care system. Manga highlighted this almost 25 years ago [90] and today, evidence suggests that closer alliances between chiropractors and medical doctors lead to improved management, reduced chronicity and enhanced patient satisfaction. [91, 92]

Secondly, integration with the mainstream represents a benefit to the economy. Recent research examining key indicators demonstrate chiropractic care to be safe, effective and cost-effective in a variety of situations, including:

- Reducing workers compensation claims [93]
- Managing sequelae of mild traumatic brain injury [94]
- Reducing workplace absenteeism [95]
- Improving outcomes in hip osteoarthritis [96]
- Reducing chest pain associated with stable angina [97]
- Reducing use of opioids for chronic pain [98]
- Reducing rates of spinal surgery [99]

Given costly surgical alternatives [100] or addictive opioids [101], increased utilisation of chiropractic services represents a proven, cost saving option for the management of musculoskeletal disease.

Finally, closer integration with the mainstream represents a long sought opportunity to contribute meaningfully to society, increase market share and enjoy increased cultural authority. The Swiss experience [92] has shown that mingling of doctors and chiropractors enhances medical referrals and social perception of chiropractors. Furthermore, it allows for the collaborative training of chiropractic students within teaching hospitals, deepening expertise and enriching learning [102].

The data emerging from Alberta Canada demonstrate that increased utilisation is achievable and sustainable. By promoting a position that chiropractic is the management of musculoskeletal disorders by conservative manual means and encouraging collaborative care, Alberta’s chiropractors enjoy a province-wide 20% utilisation rate. Chinook, a region in Alberta currently has the highest percentage of residents who have received chiropractic services (26%) and this has significantly increased from 15% in 2004. Add to these figures a 90% satisfaction rating and one might easily conclude that Albertan chiropractors are on the right track [103].

The latest data to emerge from Alberta was gathered by Janet Brown Opinion Research, between November 24 and December 22, 2016. These data show 24% of Albertans are currently seeing a chiropractor, or had seen a chiropractor in the past 12 months while 65% reported that they had received treatment from a chiropractor in the past. This is up from 58% in 2014. Further, patient satisfaction continues to be very high. Ninety-three per cent of those who are currently seeing a chiropractor provided a very satisfied rating [104].

It is worth noting that the Albertan data appear to be the highest utilization rate reported anywhere internationally in a public survey and is significantly higher than the 6–12% found by Lawrence and Meeker in their study of chiropractic utilization [105].

## A way forward?

Despite historical differences, could or should the chiropractic profession find a unified way forward? Indeed, some within the profession [50] have questioned the merit of ‘unity at any cost’ while others have gone so far as suggesting the profession be officially split along ideological lines [6]. Given fundamental differences between questioners and believers, seeking a false middle ground may only serve to increase in-fighting.

On the other hand, McGregor et al. [2] have noted a shift in the focus of many within the profession, away from divergence and towards conservative spinal care. This practice pattern appears to reflect both patients’ wants [8] and needs [106–108].

Perhaps then unity should be achieved not by arguing over the primacy of vitalism – a long out-dated and thoroughly discredited ideology [109–113] - but rather by joining with the public in addressing the need for conservative spinal care, public health, activity modification and nutritional guidance [114–116]. Practice trends suggest that chiropractors already routinely engage in these activities [117, 118] and Murphy et al. have demonstrated this as a viable way forward, suggesting podiatry as a model for change [119].

While such a reframe may alarm vitalists, it need not lead to loss of identity nor a fundamental change in job description, as Humphreys et al. [92] have shown. Instead, by rejecting the notion of vitalism and its associated metaphysical concepts (*entelechy, “elan vital”, vis essentialis,* etc.), chiropractors would be freed to focus on holism or more appropriately organicism – a paradigm that recognises that the sum of the parts is greater than the whole without invoking supernatural forces [111–113]. Further, holism, or more appropriately the biopsychosocial model of care, advocates treating of the whole person, taking into account mental and social factors, rather than just the symptoms of a disease [120]. This is a position that chiropractic has embraced since the outset [121, 122].

Such a reframe would allow chiropractors to occupy several different underserved niches in health care [17], without losing sight of the integrative nature of the body. Furthermore, it would allow the vast majority of mainstream co-aligned chiropractors to more easily collaborate with existing health care systems.

Ultimately, this form of unity would seek to unite professionals and prevent chiropractic from becoming ‘unique and extinct’. While many of the perpetuators of the schism were forces external to the chiropractic profession, the pathway to unification is under the control of the profession.

There are signs that segments of the profession are ready, willing and able to move the Palmerian ideology into the history books where it belongs. Indeed, there are clear indications that there is a move in this direction within the profession worldwide. Both the General Chiropractic Council in the United Kingdom [123] and Chiropractic Australia [124] have formally recast subluxation theory as a historical – not clinical – concept. Likewise, the International Chiropractic Education Collaboration – an alliance of leading chiropractic schools – has squarely rejected subluxation theory and instead chosen to focus on graduating students ready to embrace evidence based practice, mainstream collaboration and public health initiatives [125].

Certainly, if the Swiss, Danish and Albertan experiences have taught us anything, it is that growth is possible once dogma is dropped. Time will tell if the profession as a whole is mature enough to do so.

## Conclusion

In this paper we have examined the historical origins of the century old ideological gulf that cleaves the chiropractic profession. The schism as it is known has been identified as a division between those who adhere to the dogma of Palmerian ideology and those who embrace scientific advancement. Also considered were the some of the reasons, largely external to the profession for the perpetuation of the divide. We have demonstrated the benefits to both the society and the profession that can be achieved by progressing from the founder’s ideology and suggested a pathway to unification.

## Footnotes

1 Readers wishing greater explanations for the fallacies referred to here are invited to read: Bennett, B. (2015). Logically Fallacious: The Ultimate Collection of Over 300 Logical Fallacies (Academic Edition): eBookIt. com. or visit: https://www.logicallyfallacious.com/tools/lp/Bo/LogicalFallacies/184/The_Fallacies_Ac_An

2 For an annotated bibliographic listing of BJ Palmer’s epigrams readers are directed to: Success, Health and Happiness. The Epigrams of BJ Palmer. by Simon A Senzon. 2010, Integral Altitude Publishing, Asheville, NC.
